# Assessing unmet needs in patients with cancer: An investigation of differential item functioning of the Needs Evaluation Questionnaire across gender, age and phase of the disease

**DOI:** 10.1371/journal.pone.0179765

**Published:** 2017-07-25

**Authors:** Francesca Chiesi, Andrea Bonacchi, Caterina Primi, Guido Miccinesi

**Affiliations:** 1 Department of Neuroscience, Psychology, Drug Research, and Child Health (NEUROFARBA) -Section of Psychology, University of Florence, Italy; 2 Centro Studi e Ricerca Synthesis, Florence, Italy; 3 Cancer Research and Prevention–ISPO, Clinical and Descriptive Epidemiology Unit, Florence, Italy; IRCCS Istituto Auxologico Italiano, ITALY

## Abstract

The Needs Evaluation Questionnaire (NEQ) is a self-administered instrument used in oncology clinical practice and research.

**Objective:**

The main aim of this study was to provide evidence of the broad employability of the NEQ with patients of different gender and age with cancer in different phases of the disease and care process, using an Item Response Theory (IRT) approach and investigating Differential Item Functioning (DIF).

**Methods:**

The NEQ was completed by 762 patients visiting, consecutively, outpatient clinics or admitted to oncology wards. Patients included in the study had different primary tumor sites and were in different phases of the disease and care process. The properties of the questionnaire were analyzed by applying IRT to test how well each item of the scale concurs in measuring unmet needs, how reliable the whole scale is, and whether the scale was metrically invariant across gender, age, and phase of the disease.

**Results:**

Results showed that the NEQ performed well in measuring unmet needs and measurement equivalence of the scale across gender, age, and phase of the disease was verified.

**Conclusions:**

The current study supports the utility and broad employability of the NEQ, thus providing empirical evidence that it is psychometrically sound and metrically equivalent across different groups of cancer patients. As such, the scale could be an effective tool when planning psychosocial interventions to improve the care process and patients’ quality of life.

## Introduction

Patients with cancer have different needs which are, in part, related to the illness and in part to the care process [1–6]. As stated by Osse et al. [7] an unmet need corresponds to a desire to receive support with a demand perceived by the patient as not adequately met by the care system, and as such it is a request to the health system in general and to the staff involved in caring in any clinical setting. These needs are distributed in several main thematic areas: information and dialogue with physicians [8–11], assistance/care [12,13], psychosocial support [9,11,14,15], spiritual issues [16], and sexual problems [17,18].

Many patients still do not voluntarily express their concerns to oncologists or nurses. As far as the control of symptoms, some patients feel that suffering (pain, anxiety, depression, anger, etc.) is inevitable when you have cancer, or that there is no effective treatment to address those issues which doctors do not include spontaneously among their concerns [1]. In many cancer patients, especially in males and the elderly, low levels of spontaneous communication of unmet needs have been observed [19–21]. In oncology practice, therefore, the introduction of routine evaluation of patients’ unmet needs, based on clinical interviews and specific questionnaires, has become a common practice. Indeed, the evaluation of unmet needs of cancer patients offers oncologists individual responses and helps solve specific problems (e.g. information on the diagnosis or therapy, control of symptoms, psycho-emotional support) and, in subgroups of patients with similar socio-demographic and clinical characteristics, helps identify intervention protocols and targeted services.

To assess the unmet needs in patients with cancer several specific scales have been developed but only a few of them have been proven reliable and valid measurements [22,23]. Among them, the Needs Evaluation Questionnaire (NEQ) [8,24], a self-administered scale particularly useful both in clinical practice and research due to its agility and ease of administration. The items on the NEQ are divided into five main areas: information needs, needs related to assistance/care, relational needs, psycho-emotional needs and material needs. The scale was originally developed by Tamburini and colleagues for use in hospital oncology settings, but recently the validity of this scale with outpatients from oncology Day Hospital settings, follow up ambulatory, and rehabilitation units was supported [25].

The psychometric properties of the NEQ have been investigated according to Classical Test Theory [24, 26], a traditional and widely used approach to evaluate psychological assessment instruments. To provide further evidence of the suitability of the NEQ in measuring unmet needs in patients with cancer, in the current study we investigated the psychometric properties of the scale by employing Item Response Theory (IRT). IRT is a parametric statistical modeling procedure which involves fitting a hypothetical model to sample data, assuming that the characteristics of items on a test (i.e. item parameters) and the characteristics of individuals (i.e. latent traits) are related to the probability of a positive response (i.e. a trait-consistent endorsement of an item). Since applications of IRT have potential benefits in testing the accuracy of assessment instruments, we propose that IRT can help in testing the psychometric properties of the NEQ.

Preliminarily, we studied the characteristics of the single items and the characteristics of the whole scale. Indeed, IRT analyses provide: item location and discrimination parameters that enable evaluation of the level of the construct targeted by the item, and how well an item performs in measuring that level of the underlying construct; and the *Test Information Function* (TIF) which, instead of providing a single value (e.g. coefficient alpha) for reliability, evaluates the precision of the test at different levels of the measured construct [27, 28].

Then, since the main aim of this study was to provide evidence of the broad employability of the NEQ, our study aimed at investigating the measurement equivalence of the scale across different groups. Indeed, the NEQ has been used with patients of varying age, gender, and phase of the disease and care process (e.g. inpatients and outpatients). Nonetheless, the needs described by the items of the NEQ may be understood or interpreted differently by different respondents (e.g. the readability of the items might be greater for younger than older people, the meaning of the items might change for men or women, the appropriateness of the vocabulary might not be the same for patients during diagnosis and/or treatment or patients during follow up). As such, testing the invariance of the NEQ across these groups might be especially useful to ensure valid interpretation of scores as well as group differences. This fundamental measurement issue is adequately addressed by IRT procedures that allow the assessment of *Differential Item Functioning* (DIF) [27, 29], which examines the relationship between item response and another variable, called the group variable (e.g. gender). This latter depends on measurement of an underlying construct (e.g. unmet needs) to ascertain whether, after controlling for the underlying construct, the response to an item is related to group membership. For example, a randomly selected woman with a specific unmet need and a randomly selected man with the same unmet need should have the same chance of endorsing an item referring to this need. If this not the case, the item is biased. Thus, DIF analysis involves three factors: item response, trait level, and subgroup membership. If a test includes many items with DIF, the differences in the scores are not an exclusive function of the measured trait but there are some artifacts in the measurement process due to group membership.

To sum up, in order to provide evidence of the suitability of the NEQ in measuring unmet needs in patients with cancer we investigated: how well each item of the scale concurs in measuring unmet needs, how reliable the whole scale is in measuring unmet needs, and whether items have different measurement properties in different groups testing the equivalence of the NEQ items across gender, age, and phase of the disease.

## Materials and methods

### Participants

The present sample is part of a wider survey on unmet needs of cancer patients called I.B.I.S. (Indagine sui Bisogni Insoddisfatti nella Sanità) Project [Survey on Unmet Health Care Needs]. The survey involved patients from six different oncology units in Tuscany, Italy. Participation was proposed to all patients visiting, consecutively, outpatient clinics or admitted to oncology wards, regardless of site or stage of the tumour. The percentage of patients who accepted to take part in the research in the different medical units ranged from 71.0% to 95.4%.

Clinical data and, in particular, phase of disease and care process were provided by oncologists. Exclusion criteria were: age under 18 or over 90, cognitive impairment or psychiatric diseases symptoms, severe symptoms due to illness or side effects of therapy that precluded to complete questionnaires independently. The total sample was composed of 762 patients (65% female) with a mean age of 61.71 years (*SD* = 12.06).

In DIF analyses, the male group was composed of 267 patients (*M*_*age*_ = 64.85, *SD* = 12.36; range 18–90), and the female group was composed of 493 patients (*M*_*age*_ = 60.02, *SD* = 11.56; range 23–90). For age, we created two groups: Under 65 (*N* = 416, 73% females, *M*_*age*_ = 53.00, *SD* = 8.69; range 18–64) and Over 65 (*N* = 343; 55% females, *M*_*age*_ = 72.26, *SD* = 5.17; range 65–90). For the phase of the disease, we compared two groups: Diagnosis/Treatment (*N* = 297, 53% females, *M*_*age*_ = 60.99, *SD* = 12.22; range 18–90) and Follow Up/Rehabilitation (*N* = 291; 85% females, *M*_*age*_ = 61.30, *SD* = 11.86; range 18–85). The remaining participants belonged to the following groups: Relapse/recurrence (*N* = 40), Progression of the disease (*N* = 41), and Palliative care (*N* = 15). Due to the small sample size, these groups were excluded from the analysis as well as 78 patients of which information about the phase of the disease were not available.

The study received approval from local ethics committees: Comitato Etico Locale Azienda Ospedaliero-Universitaria Careggi, Firenze; Comitato Etico Locale Azienda Unità Sanitaria Locale 10, Firenze; Comitato Etico Locale Azienda Unità Sanitaria Locale 4, Prato, Comitato Etico Locale Azienda Unità Sanitaria Locale 1, Massa Carrara. Patients received an informational brochure about the study and were asked to provide written informed consent.

### Measure and procedure

Participants filled out a paper-and-pencil self-report battery which included the NEQ [8,24], a self-administered instrument with 23 dichotomous items (i.e. yes/no answer) assessing needs in five areas: informative needs, needs related to assistance /care, relational needs, needs for psycho-emotional support, material needs (see Appendix). This battery was presented by the psycho-oncologist or by nurses or physicians, depending on the setting (ward, day hospital, rehabilitation ambulatory or follow up ambulatory), the second day after admission to the ward or during waiting periods at the day hospital or ambulatory. Patients were assured that participation was totally free and voluntary and that non-adherence did not alter care received by ward staff. They did not receive any assistance in completing the battery. Approximately, they employed a time of between 5 and 10 min to answer the NEQ.

### Statistical analyses

To apply IRT modeling it is important to ascertain if there is a common factor among the items; if so, item parameters reflect the relation between the common latent trait and the item responses [30–32]. Since the NEQ has been described as multidimensional [24–26] -due to clusters of items that reflect specific needs (i.e. needs belonging to the same domain)- we started testing if the scale can be considered “unidimensional enough” so that the item parameter estimates properly reflect the latent trait held in common among the items (i.e. unmet needs). Indeed, the robustness of unidimensional IRT model parameter estimates to multidimensionality violations has been demonstrated, concluding that if the data have a “strong” common factor or multiple highly correlated factors, then IRT item parameter estimates are not seriously distorted [30,31]. Thus, to verify this prerequisite we tested a second-order model (i.e. we tested the hypothesis that seemingly distinct but related constructs can be accounted for by one common underlying higher order construct) in which the higher-order latent variable was the general unmet needs whose influence is shared among the five first-order specific needs (information needs, needs related to assistance/care, relational needs, psycho-emotional needs and material needs) as defined by Annunziata et al. [26]. This Confirmatory Factor Analysis (CFA) for dichotomous data was implemented in Mplus software [33].

The goodness of fit of the IRT model was evaluated using M_2_ statistic and the associated root mean square error of approximation (RMSEA) value. M_2_ statistic, like other chi-square statistics, is generally unrealistic because there will be some error in any strong parametric model, thus the RMSEA provides a metric for model error [34]. The item fit under the IRT model was tested for each item computing the S-χ^2^ statistics. Due to the large sample size and the above mentioned limitations of the chi-square statistics, α was fixed at .01. IRT models use the original response data to estimate probabilities of responses as a function of the latent trait (*theta*). Item parameters are: the location parameters (*b*), which indicate the trait level where there is a 0.5 probability of endorsing the affirmative option, and the discrimination parameter (*a*), which indicates the ability of an item to discriminate people of different levels of the underlying trait.

We investigated the *Test Information Function* (TIF) which provides test reliability estimations, indicating the precision of the whole test for each level of the latent trait [27]. This means that the more information the test provides at a particular ability level, the smaller the error associated with ability estimation is and thus the higher the test’s reliability. In terms of graphical presentation, the test information curve shows how well the construct is measured at different levels of the underlying measured trait.

To test the measurement invariance of the NEQ across genders, ages, and phases, DIF analyses were performed. The DIF detection procedure is based on a nested model comparison approach. First, a more parsimonious model is tested with all parameters constrained to be equal across groups for a studied item against an augmented model. Here, one or more parameters of studied item are freed to be estimated distinctly for the two groups (a focal group and a reference group). This procedure involves comparing differences in log-likelihoods (distributed as chi-square) associated with nested models. To adjust for multiple comparisons and the aforementioned limitations of the chi-square statistics, α was fixed at .01. Initial DIF estimates can be obtained by treating each item as a studied item while using the rest as “anchor” items. Anchor items are assumed without DIF and are used to estimate the trait, and to link the two groups being compared in terms of trait levels. Anchor items are selected through a process of log-likelihood comparison performed iteratively and called “purification” procedure. During this iterative process the DIF status of items may change as a result of using a less than optimal conditional variable at various steps in the analyses. Since DIF analyses examine differences in item parameters, two types of DIF can be detected: uniform DIF that refers to location parameters, and non-uniform DIF that refers to discrimination parameters.

All IRT analyses were conducted employing IRTPRO software [35].

## Results and discussion

The second-order model showed a goof fit (CFI = .96; TLI = .98; RMSEA = .055). The factor loadings were all significant (*p* < .01) and adequate in size, ranging from .43 to .94. The first-order latent variables were all significantly related to the second order latent variable and second-order loadings ranged from .77 to .98.

The unidimensional IRT model showed satisfactory fit (M_2_ = 1144.55, *df* = 230, *p* = 0.0001; RMSEA = .07). Each item had a non-significant S-χ^2^ value, indicating that all items fit under unidimensional IRT model. Item calibration showed that all the *b* values were above the mean trait level with the exception of item 2 which was slightly below the mean. Specifically, as showed in Table 1, eleven items were about a half standard deviation above the mean, four items about one, and the remaining eight items one and a half standard deviations or more above the mean. In this case, higher values identify needs perceived to a lesser extent (e.g. Item 16: “I need economic help”), while lower values represent compelling needs (e.g. Item 2: “I need more information about my future condition”). The range of item discrimination parameters was between .65 and 3.90. According to Baker [36], seven items showed moderate, five high, and the remaining eleven items very high discriminative power.

**Table 1:** Discrimination and location parameters for each item of the Needs Evaluation Questionnaire (NEQ).

*Item*	*α*	*b*	*Item*	*a*	*b*
*1*	2.26	0.42	*13*	2.40	0.50
*2*	1.92	-0.11	*14*	1.01	1.00
*3*	3.90	0.35	*15*	1.33	0.40
*4*	3.19	0.22	*16*	0.65	2.62
*5*	2.56	0.49	*17*	0.93	1.57
*6*	3.47	0.48	*18*	0.93	2.15
*7*	2.89	0.61	*19*	0.73	0.65
*8*	2.57	0.35	*20*	1.44	1.15
*9*	1.72	0.48	*21*	1.33	0.88
*10*	1.01	2.55	*22*	1.56	1.11
*11*	1.58	1.38	*23*	0.90	1.33
*12*	2.08	1.42			

*a* = discrimination parameter; *b* = location parameter.

**Fig 1:**
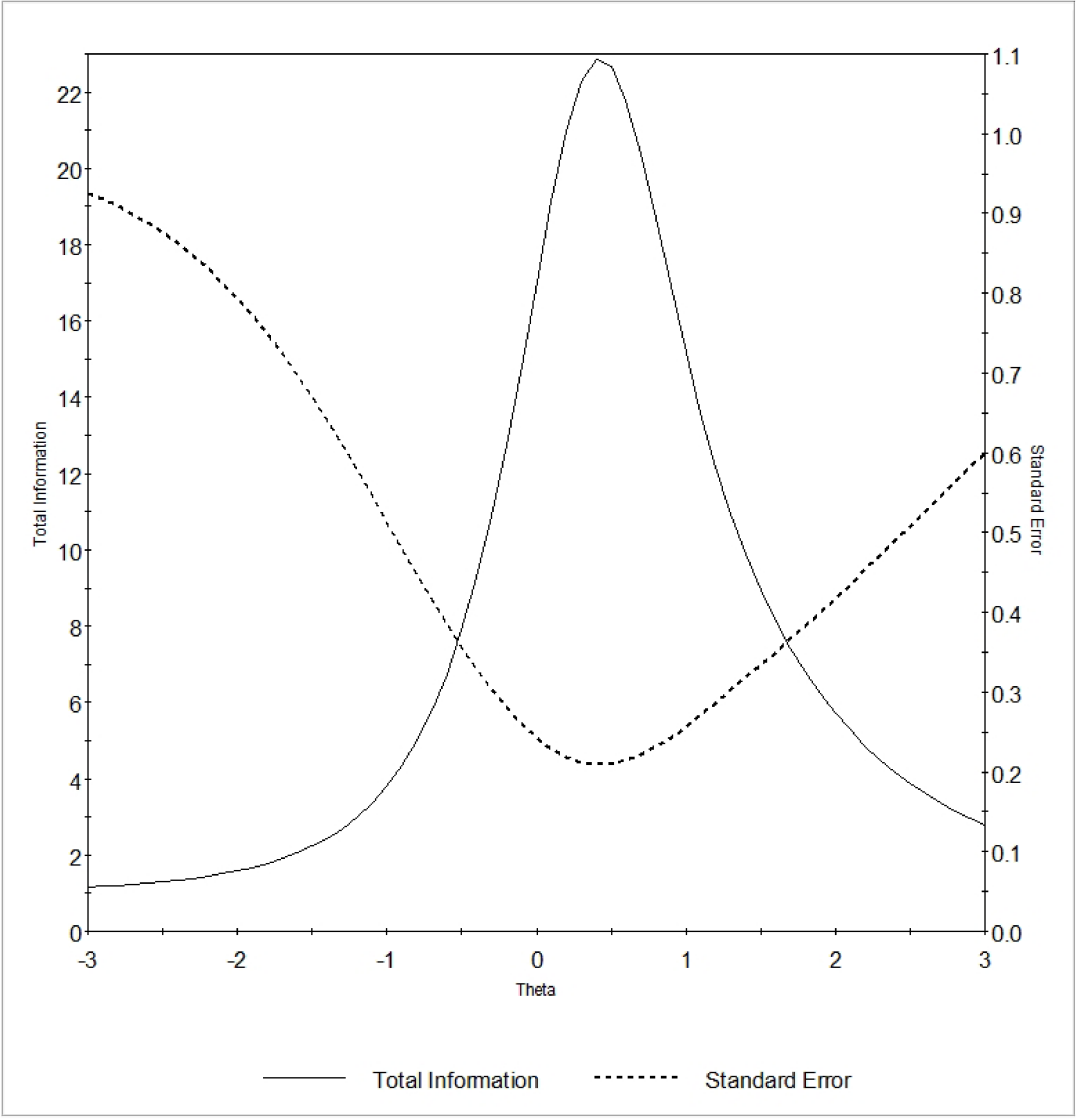
Test Information Function of the *Needs Evaluation Questionnaire* (NEQ). Latent trait (Theta) is shown on the horizontal axis. The amount of information (solid line) and the standard error (dotted line) yielded by the test at any trait level are shown on the vertical axis.

The TIF (Fig 1) showed that within a large range of trait, the amount of test information was equal to or greater than 4 indicating that the instrument was sufficiently informative for this range of the trait. Indeed, if we interpret the information magnitude by computing the associated reliability (*r* = 1-1/Information), reliability was equal to or greater than .75 within the aforementioned range.

In the first step of gender DIF analyses (in which the male group was the reference group), item 18 was identified as the studied item (*p* = .0013). Then, using all the other items as “anchor” items, the analysis was repeated. During this iterative process the DIF status of item 18 did not change. Specifically, whereas discrimination parameters were invariant across groups, this item showed uniform DIF referring to location parameters (i.e. male respondents were consistently less likely than female respondents to endorse this item). Nevertheless, since just one item out of 23 exhibits DIF, the NEQ can be considered invariant across gender.

Concerning age, comparing Under 65 (reference group) and Over 65, no item exhibited DIF from the first step of the analysis. As such, the NEQ can be considered invariant across age.

For the phase of the disease, comparing Diagnosis/Treatment (reference group) and Follow Up/Rehabilitation, item 10 was identified as the studied item (*p* = .0077). Then, using all the other items as “anchor” items, the analysis was repeated. During this iterative process the DIF status of item 10 did not change. Specifically, whereas discrimination parameters were invariant across groups, this item showed uniform DIF referring to location parameters (i.e. Follow Up/Rehabilitation patients were consistently less likely than Diagnosis/Treatment patients to endorse this item). Again, since just one item exhibits DIF, the NEQ can be considered invariant across the phase of the disease.

**Fig 2:**
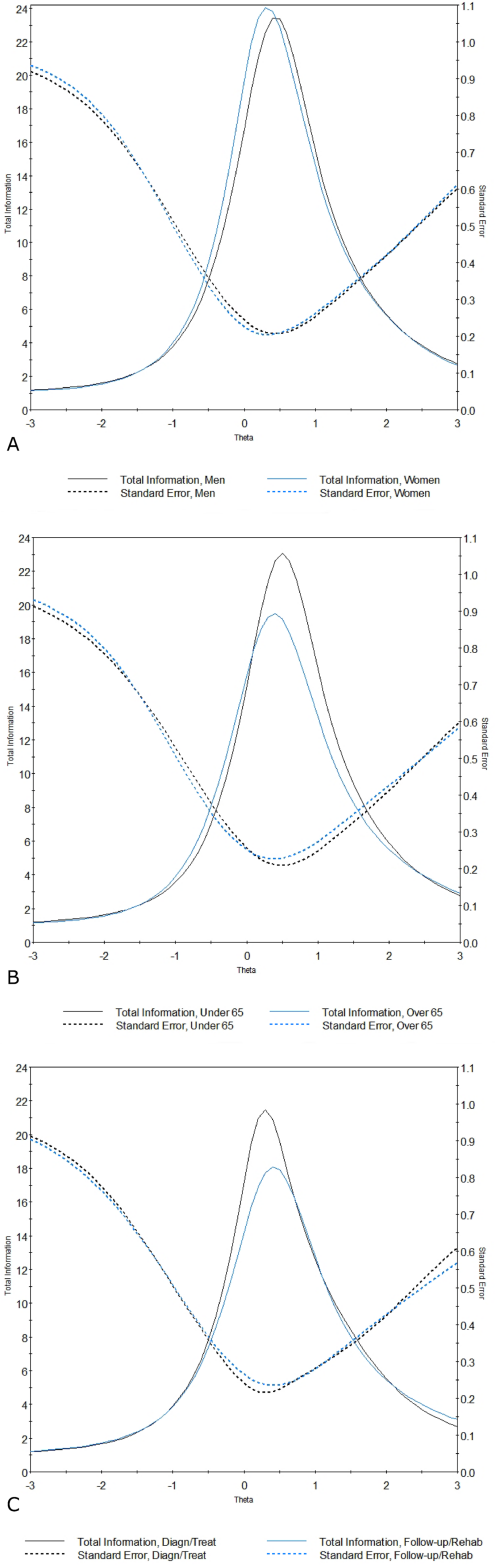
**Test Information Function of the *Needs Evaluation Questionnaire* (NEQ) across gender (a), age (b), and phase of the disease (c).** Latent trait (Theta) is shown on the horizontal axis. The amount of information (solid line) and the standard error (dotted line) yielded by the test at any trait level are shown on the vertical axis.

From visual inspection of the TIFs of the different groups, we can see that the scale functions exactly in the same way across groups (Fig 2). Indeed, the amount of test information was sufficiently high within the same range of the trait, and the standard errors of the measurement were very similar attesting to measurement equivalence of the scale.

## Conclusion

The NEQ was originally developed by Tamburini and colleagues [8,24] for use in oncology wards to assess the needs of cancer patients and their families. Indeed, patients diagnosed with cancer have many needs with regard to relief from physical and psycho-social distress and to improve their quality of life. Some of the most important needs concern the possibility to be cured or to have their life prolonged, the control of symptoms, information and dialogue with clinicians, and material, psychological and spiritual support [1–4]. Therefore, the introduction of a routine and customized assessment of the unmet needs of cancer patients has become very common. As such, it is important that the instrument employed to assess the needs be psychometrically sound and equally suitable for patients with different characteristics, e.g. men or women, old or young, inpatients or outpatients in oncology settings. For these reasons, the current paper aims to provide evidence of the suitability of the NEQ in measuring unmet needs investigating whether the scale is metrically invariant across gender, age, and phase of the disease, and how reliable the whole scale is across these groups.

Overall, the spread of location parameters indicated that the described needs were perceived differently. Additionally, all the items have an adequate discriminative power, i.e. able to distinguish among people who have or do not have each specific need described by the item. With regard to reliability, the scale was sufficiently informative for a large range of the trait. This result might be indicative of the utility of the NEQ in recognizing among patients with cancer those who truly have a significant number of unmet needs in order to care for them and promote intervention strategies.

The NEQ appears to perform equally well regardless of gender, age, and phase of the disease of patients with cancer. Indeed, with only two exceptions, the items on the scale did not show a different functioning across groups. With regard to reliability of the whole scale, the NEQ was equally informative for quite a large range of the traits in these different subsamples. This finding confirms that the meaning, the wording, and the readability of the items are the same for men and women, younger and older patients, patients during diagnosis and/or treatment or patients during follow up and/or rehabilitation. As such, the scale ensures valid interpretation of scores as well as of group differences regardless of the specificity of the patient’s characteristics.

Due to its agility and ease of administration the scale has been proven to be particularly useful both in clinical practice and research [8,24,25]. The current study supports the utility and large employability of the scale, providing empirical evidence that the NEQ is psychometrically sound and metrically equivalent across different groups. Moreover, the scale assesses with adequate reliability the measured construct effectively distinguishing individuals with different levels of unmet needs. A practical consequence of this is that the scale can be used to detect patients who need support so they may receive targeted effective answers (e.g. information on diagnosis or therapy, control of symptoms, psycho-emotional support). Additionally, no relevant differences were found in the item functioning when comparing men and women, older and younger respondents, and patients in a different phase of the disease. As a result, no different scoring rules or interpretation are needed when using the scale with different populations, and it can be used in subgroups of patients with different demographic and clinical characteristics inside wide intervention protocols and targeted services.

However, this research has some limitations which must be noted. First, there are some problems in generalizing the obtained findings since we employed an Italian sample. Moreover, due to the small sample sizes, differences among some particular subsamples (e.g. relapse) or extremely different groups (e.g. very young vs very old patients) were not investigated. Arguably, it would be noteworthy to investigate if scale functions differently for some kinds of patients, at least for some items/needs. Future studies might confirm and extend the current results and go beyond the limitations of the present findings. To extend the use of the current scale it would be relevant to test the psychometric properties of different language versions of the scale in different oncology settings. In the same way, DIF analysis across language and settings should be performed to provide additional evidence of the invariance property of the scale. As such, the NEQ might be a robust assessment that provides comparable scores from different contexts. Finally, to provide evidence of the suitability of the NEQ in test-retest research design (e.g. in studies aiming at verifying the efficacy of intervention programs on patient’s needs), it would be relevant to test the stability of the instrument over time.

In conclusion, the NEQ seems to be an effective tool across different age, gender, and phases of the disease, and care process patients in the assessment of unmet needs.

## Appendix

### Needs Evaluation Questionnaire

The questions listed below regard your current needs and state of mind. Don't overthink your answers; usually your first reaction is the most authentic one.

1. I need more information about my diagnosis
2. I need more information about my future condition
3. I need more information about the exams I am undergoing
4. I need more information about the treatments
5. I need to be more involved in the therapeutic choices
6. I need clinicians and nurses to give me information which is easier to understand
7. I need clinicians to be more honest with me
8. I need to be able to talk more with the doctors
9. I need some of my symptoms (pain, nausea, insomnia, etc.) to be better controlled
10. I need more help with eating, dressing, and going to the bathroom
11. I need more respect of my privacy
12. I need to be treated with more respect by the nursing staff
13. I need to be reassured more by the doctors
14. I need the hospital to provide better services (i.e. bathrooms, food service, cleanliness)
15. I need more financial/insurance information regarding my illness
16. I need economic help
17. I need to speak with a psychologist
18. I need to speak with a spiritual guide
19. I need to speak with people who have had my same experiences
20. I need more reassurance from my relatives
21. I need to feel more useful in my family
22. I need to feel less left on my own
23. I need to feel less pitied by other people

What other particular needs do you have in this moment?

## Supporting information

S1 File This is the NEQdataset. (DAT)

S2 File This is the NEQdataset scoring. (DOC)

## References

[1] Wen KY, Gustafson DH. Needs assessment for cancer patients and their families. Health Qual Life Outcomes. 2004;2: 11; doi: 10.1186/1477-7525-2-11 14987334 10.1186/1477-7525-2-11 PMC394345

[2] Baum M. What are the needs of patients diagnosed with cancer? Psycho-Oncology. 2004;13: 850–852.

[3] Carey M, Lambert S, Smits R, Paul C, Sanson-Fisher R, Clinton-McHarg T. The unfulfilled promise: a systematic review of interventions to reduce the unmet supportive care needs of cancer patients. Supp Care Cancer. 2012;20: 207–219. 10.1007/s00520-011-1327-1 PMC3244607 22089430

[4] Harrison JD, Young JM, Price MA, Butow PN, Solomon MJ. What are the unmet supportive care needs of people with cancer? A systematic review. Supp Care Cancer. 2009;17: 1117–1128. 10.1007/s00520-009-0615-5 19319577

[5] Fitch M. Supportive care for cancer patients. Hosp Q. 2000;3: 39–46. 11482268 10.12927/hcq..16542

[6] Sanson-Fisher R, Girgis A, Boyes A, Bonevski B, Burton L, Cook P. The unmet supportive care needs of patients with cancer. Supportive Care Review Group. Cancer. 2000;88: 226–237. 10618627 10.1002/(sici)1097-0142(20000101)88:1<226::aid-cncr30>3.3.co;2-g

[7] Osse BH, Vernooij-Dassen MJ, de Vree BP, Schadé E, Grol RP. Cancer. Assessment of the need for palliative care as perceived by individual cancer patients and their families: a review of instruments for improving patient participation in palliative care. Cancer. 2000;88: 900–911. 10679661 10.1002/(sici)1097-0142(20000215)88:4<900::aid-cncr22>3.0.co;2-2

[8] Tamburini M, Gangeri L, Brunelli C, Boeri P, Borreani C, Bosisio M, et al Cancer patients’ needs during hospitalization: a quantitative and qualitative study. BMC Cancer. 2003;23: 12. 10.1186/1471-2407-3-12 PMC155542 12710890

[9] Willems RA, Bolman CA, Mesters I, Kanera IM, Beaulen AA, Lechner L. Cancer survivors in the first year after treatment: the prevalence and correlates of unmet needs in different domains. Psycho-Oncology. 2016; 25: 51–57; doi: 10.1002/pon.3870 26110652 10.1002/pon.3870

[10] Girgis A, Boyes A, Sanson-Fisher RW, Burrows S. Perceived needs of women diagnosed with breast cancer: rural versus urban location. Aust NZ J Public Health. 2000;24: 166–173. 10.1111/j.1467-842x.2000.tb00137.x 10790936

[11] Sutherland G, Hill D, Morand M, Pruden M, McLachlan SA. Assessing the unmet supportive care needs of newly diagnosed patients with cancer. Eur J Cancer Care. 2009;18: 577–584. 10.1111/j.1365-2354.2008.00932.x 19549286

[12] Davis C, Williams P, Redman S, White K, King E. Assessing the practical and psychosocial needs of rural women with early breast cancer in Australia. Soc Work Health Care. 2003;36: 25–36. 12564650 10.1300/j010v36n03_02

[13] Soothill K, Morris SM, Harman J, Francis B, Thomas C, McIllmurray MB. The significant unmet needs of cancer patients: probing psychosocial concerns. Supp Care Cancer 2001;9: 597–605. doi: 10.1007/s005200100278 10.1007/s005200100278 11762970

[14] Clavarino AM, Lowe JB, Carmont S-A, Balanda K (2002) The needs of cancer patients and their families from rural and remote areas of Queensland. Aust J Rural Heal. 2002;10: 188–195. doi: 10.1046/j.1440-1584.2002.00436.x 10.1046/j.1440-1584.2002.00436.x 12121408

[15] Bonacchi A, Rossi A, Bellotti L, Franco S, Toccafondi A, Miccinesi G, Rosselli M. Assessment of psychological distress in cancer patients: a pivotal role for clinical interview. Psycho-Oncology. 2010;19: 1294–1302. doi: 10.1002/pon.1693 20148442 10.1002/pon.1693

[16] Nixon A, Narayanasamy A. The spiritual needs of neuro-oncology patients from patients' perspective. J Clin Nurs. 2010;19: 2259–2370. doi: 10.1111/j.1365-2702.2009.03112.x 20529167 10.1111/j.1365-2702.2009.03112.x

[17] Lintz K, Moynihan C, Steginga S, Norman A, Eeles R, Huddart R, Dearnaley D, Watson M. Prostate cancer patients’ support and psychological care needs: survey from a non-surgical oncology clinic. Psycho-Oncology. 2003;12: 769–783. doi: 10.1002/pon.702 14681951 10.1002/pon.702

[18] Steginga SK, Occhipinti S, Dunn J, Gardiner RA, Heathcote P, Yaxley J. The supportive care needs of men with prostate cancer. Psycho-Oncology. 2001;10: 66–75. 11180578 10.1002/1099-1611(200101/02)10:1<66::aid-pon493>3.0.co;2-z

[19] Cassileth BR, Zupkis RV, Sutton-Smith K, March V. Information and participation preferences among cancer patients. Ann Intern Med. 1980;92: 832–836. 7387025 10.7326/0003-4819-92-6-832

[20] Lupton D. Your life in their hands: trust in the medical encounter In: James V, Gave J eds Health and the sociology of emotions. Oxford: Blakwell; 1996 pp. 57–72

[21] Harrison J, Maguire P, Pitceathly C. Confiding in crisis: gender differences in pattern of confiding among cancer patients. Soc Sci Med. 1995;41: 1255–60. 8545678 10.1016/0277-9536(94)00411-l

[22] Richardson A, Medina J, Brown V, Sitzia J. Patients' needs assessment in cancer care: a review of assessment tools. Supp Care Cancer. 2007;15: 1125–1144. 10.1007/s00520-006-0205-8 17235503

[23] Carlson LE, Waller A, Mitchell AJ. Screening for distress and unmet needs in patients with cancer: review and recommendations. J Clin Onc. 2012; doi: 10.1200/JCO.2011.39.5509 22412146 10.1200/JCO.2011.39.5509

[24] Tamburini M, Gangeri L, Brunelli C, Beltrami E, Boeri P, Borreani C, et al Assessment of hospitalized cancer patients’ needs by the Needs Evaluation Questionnaire. Ann Oncol. 2000;11: 31–37. 10690384 10.1023/a:1008396930832

[25] Bonacchi A, Fazzi L, Toccafondi A, Cantore M, Mambrini A, Muraca MG, et al Use and perceived benefits of complementary therapies by cancer patients receiving conventional treatment in Italy. J Pain Symptom Manage. 2014;47: 26–34. doi: 10.1016/j.jpainsymman.2013.03.014 23916679 10.1016/j.jpainsymman.2013.03.014

[26] Annunziata MA, Muzzatti B, Altoè G. A contribution to the validation of the Needs Evaluation Questionnaire (NEQ): a study in the Italian context. Psycho-Oncology. 2009;18: 549–53 doi: 10.1002/pon.1445 19021128 10.1002/pon.1445

[27] Embretson SE, Reise S P. Item response theory for psychologists Mahwah, NJ: Lawrence Erlbaum; 2000.

[28] Hambleton RK, Swaminathan H, Rogers HJ. Fundamentals of item response theory Newbury Park, CA: Sage Publications, Inc; 1991.

[29] Reise SP, Waller NG. Item response theory and clinical measurement. Annu Rev Clin Psychol. 2009;5: 27–48. doi: 10.1146/annurev.clinpsy.032408.153553 18976138 10.1146/annurev.clinpsy.032408.153553

[30] Drasgow F, Parsons CK. Application of unidimensional item response theory models to multidimensional data. Appl Psychol Meas. 1983;7: 189–199.

[31] Reckase MD. Unifactor latent trait models applied to multifactor tests: Results and implications. J Educ Behav Stat. 1979;4: 207–230.

[32] Reise SP, Moore TM, Haviland MG. Bifactor models and rotations: Exploring the extent to which multidimensional data yield univocal scale scores. J Pers Assess. 2010;92: 544–559. doi: 10.1080/00223891.2010.496477 20954056 10.1080/00223891.2010.496477 PMC2981404

[33] Muthén LK, Muthén BO. Mplus: The comprehensive modeling program for applied researchers User’s guide (3rd ed.). Los Angeles: Muthén & Muthén; 2004.

[34] Cai L. Maydeu‐Olivares A, Coffman D L, Thissen D. Limited‐information goodness‐of‐fit testing of item response theory models for sparse 2P tables. Br J Math Stat Psychol. 2006;59: 173–194. doi: 10.1348/000711005X66419 16709285 10.1348/000711005X66419

[35] Cai L, Thissen D, du Toit SHC. IRTPRO 2.1 for Windows. Chicago, IL: Scientific Software International; 2011.

[36] Baker F. The basics of item response theory ERIC clearinghouse on assessment and evaluation. MD: University of Maryland College Park; 2001.

