# Supplementary material for: Assessing unmet needs in patients with cancer: An investigation of differential item functioning of the Needs Evaluation Questionnaire across gender, age and phase of the disease
Source: PLoS One. 2017 Jul 25;12(7):e0179765. doi: 10.1371/journal.pone.0179765 (PMC5526559; doi:10.1371/journal.pone.0179765)
Supplement: S2 File — (DOC) [file pone.0179765.s002.doc]

**NEQ_1 – NEQ_23**: yes = 1: no = 0
